# RBBP6 and cancer: from molecular mechanisms to clinical implications

**DOI:** 10.3389/fonc.2026.1841906

**Published:** 2026-05-19

**Authors:** Philemon N. Ubanako, Ekene E. Nweke, Zanele Nsingwane, Bernice Monchusi, Clement Penny

**Affiliations:** 1 Molecular and Cellular Oncology Research Group, Department of Internal Medicine, Faculty of Health Sciences, University of the Witwatersrand, Johannesburg, South Africa; 2 Department of Surgery, Faculty of Health Sciences, University of the Witwatersrand, Johannesburg, South Africa; 3 Department of Life and Consumer Sciences, University of South Africa, Johannesburg, South Africa; 4 Wits Diagnostic and Innovation Hub, Wits Health Consortium, University of the Witwatersrand, Johannesburg, South Africa; 5 Department of Biosciences, Council for Scientific and Industrial Research (CSIR), Pretoria, South Africa

**Keywords:** biomarker, cancer, carcinogenesis, gene regulation, molecular mechanisms, posttranslational modifications, RBBP6, ubiquitin ligase

## Abstract

Retinoblastoma binding protein 6 (RBBP6) is a 200 kDa E3 ubiquitin ligase that performs several biological functions and plays a role in tumorigenesis. RBBP6 is overexpressed in several types of tumors, including lung, colorectal, breast, cervical, hepatocellular, and esophageal cancers, suggesting its association with malignant progression. Moreover, this increased expression has been associated with clinicopathological parameters in lung, colorectal, cervical, and ovarian cancers. This review captures the molecular functions of RBBP6 and the mechanisms by which aberrant RBBP6 expression and function influence cancer development and therapeutic response. These mechanisms include its ability to interact with E3 ligase substrates, Jun N-terminal kinase (JNK), noncoding RNAs, and DNA damage-associated proteins to promote cell proliferation, stemness, cell cycle progression, and metastasis. We also evaluated the prognostic and predictive significance of RBBP6 as a potential cancer biomarker and its suitability as a molecular target in cancer. This is the first review that comprehensively explores the multifaceted molecular mechanisms by which RBBP6 influences cancer progression in several cancer types.

## 1 Introduction

Retinoblastoma binding protein 6 (RBBP6) is a 200 kDa mRNA splicing-associated protein with E3 ubiquitin ligase activity conferred by a RING finger domain (1). RBBP6 functions include ubiquitination, mRNA processing, cell cycle control, apoptosis, and DNA replication (2–5). Loss of RBBP6 leads to early embryonic lethality in mice, emphasizing its critical role in cell viability and development. RBBP6 interacts with two key tumor suppressor proteins, p53 and retinoblastoma protein (Rb) (6, 7), and promotes carcinogenesis in several types of cancer (3, 8–12).

Recent studies revealed intriguing molecular mechanisms involved in RBBP6-mediated carcinogenesis in several types of cancer, which could pave the way for the development of RBBP6-targeted anticancer therapeutics. These include its ability to ubiquitinate novel protein substrates such as NF-kappa-B inhibitor alpha (IκBα), junction plakoglobin (JUP), and cleavage and polyadenylation specificity factor 3 (CPSF3) and tag them for proteasomal degradation (3, 11, 13). RBBP6 also interacts with noncoding RNAs such as microRNAs (miRNAs), long noncoding RNAs (lncRNAs), and circular RNAs (circRNAs), which play crucial roles in gene regulation, cellular function, and disease progression (8, 13, 14). Moreover, RBBP6 has recently been reported to promote cervical and prostate cancer through the activation of c-Jun NH2-terminal kinase (JNK) signaling. RBBP6 was reported to increase JNK phosphorylation and to stimulate cell viability, migration, and proliferation in cervical cancer. In prostate cancer, knockdown of RBBP6 induced G2/M cell cycle arrest, suppressed invasion and metastasis, and downregulated matrix metalloproteases-2 (MMP-2 and MMP-9) (15, 16). RBBP6 regulates posttranscriptional gene expression via alternative polyadenylation events and posttranslational protein modifications by its E3 ubiquitination ligase activity, thereby exhibiting a crucial role in cancer development and therapeutic response. This review explores the molecular functions of RBBP6 and the mechanisms by which it exerts its cancer-promoting effects through diverse proteins and noncoding RNAs.

## 2 RBBP6 protein structure and isoforms

### 2.1 RBBP6 structure

The *RBBP6* gene is located on chromosome 16p11.2−p12 and encodes a multidomain protein with the following domains: an N-terminal DWNN, ZnF, RING, proline-rich region, CC motif, RS domain, and p53 binding domain. The DWNN, which structurally resembles ubiquitin, regulates protein stability and interactions, and thus plays a crucial role in the cell cycle and apoptosis by modulating p53-associated pathways (1, 17, 18). Dysregulation of the DWNN domain can compromise tumor suppressor functions, resulting in oncogenesis. The RING finger domain facilitates p53 ubiquitination and tags it for degradation (19, 20). In turn, disruption of this function impairs p53 and leads to tumor progression. The p53-binding domain directly binds p53 and enhances its ubiquitination through the E3 ligase activity of RBBP6, thereby regulating apoptosis and cell cycle arrest (16, 21, 22).

The pRb-binding domain interacts with the tumor suppressor pRb to control cell cycle progression (23, 24), and its dysregulation may result in uncontrolled proliferation. Knockdown of RBBP6 isoform 3 decreased the percentage of cells in G2/M, whereas the same effect was not observed when RBBP6 isoform 1 was knocked down (17). The nuclear localization signal located in the intrinsically disordered region directs RBBP6 to the nucleus, particularly to nuclear speckles (25, 26). Finally, the arginine/serine-rich domain is involved in RNA splicing and processing (27). These structural domains highlight the pivotal role of RBBP6 in cellular homeostasis and its contributions to cancer development.

### 2.2 RBBP6 isoforms

**Figure 1:**
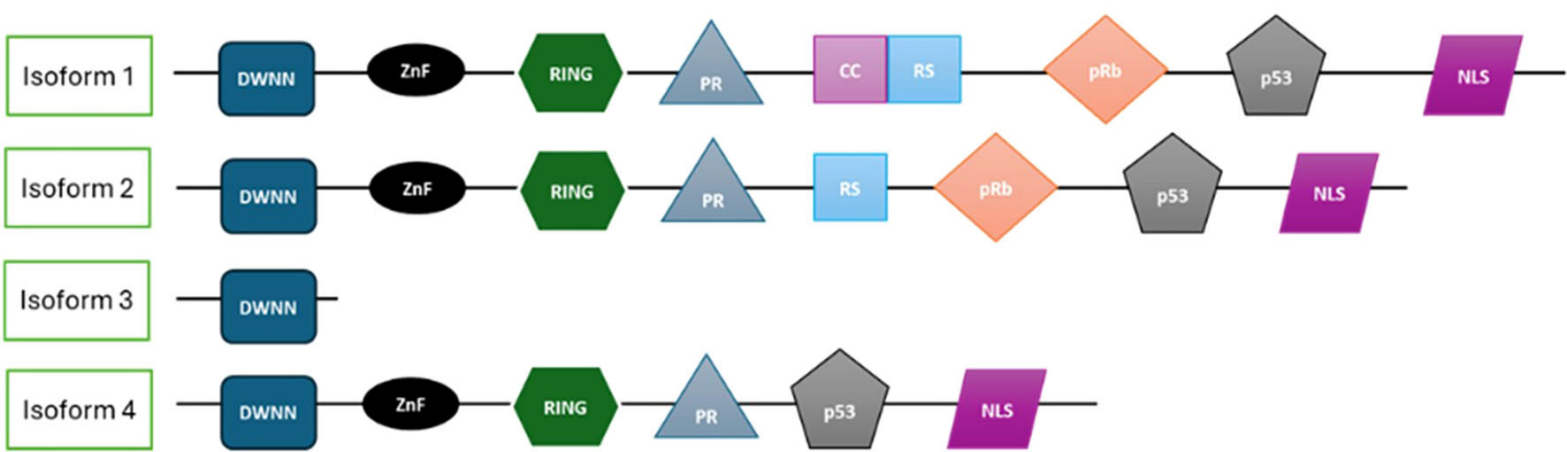
Structure of the four isoforms of RBBP6, showing the specific domains present in each. The full-length isoform 1 contains all major domains, whereas the other isoforms are progressively truncated, resulting in the loss of specific functional domains. This image was created using Microsoft Office PowerPoint.

RBBP6 encodes four human isoforms generated by alternative splicing, which possess distinct domain structures that determine their specific functional roles. The two main isoforms most characterized are isoforms 1 and 3 (28). Isoform 1 (full-length isoform), which contains all functional domains, is known to associate with tumor suppressors such as p53 and pRB, and functions as a cancer−associated isoform with pro−tumorigenic functions (1). Isoform 1 also regulates the stability of p53 through its role as a ubiquitin ligase, preventing cell cycle control, apoptosis, and important downstream events (18). Isoform 2 retains the same annotated domains as Isoform 1, but its function has not been described in peer−reviewed research. Isoform 3 consists solely of the DWNN domain and has been shown to modulate mRNA 3′−end processing and counteract the activity of the full−length protein (1, 17, 29). Isoform 3 encodes a 13 kDa variant (the shortest recombinant form) containing only the DWNN domain (**Figure 1**). Isoform 3 has been shown to play a role in cell cycle regulation and is downregulated in several cancers (17). Isoform 3, which consists only of the DWNN domain, functions as a tumor suppressor by promoting apoptosis (1). Recently, RBBP6 isoform 3 was shown to be downregulated in several cancers and inversely correlated with isoform 1 during cancer progression. Isoform 3 (DWNN) suppresses RBBP6-mediated 3’ mRNA processing by competing with RBBP6 for binding to the core mRNA processing machinery (29). These opposing functions of Isoforms 1 and 3 suggest a novel mechanism for regulating mRNA processing. The least studied isoform predicted by bioinformatic analysis is isoform 4, which lacks an RB-binding domain (18). Isoform 4 lacks the Rb−binding region, but no functional characterization has been reported in published studies. Isoform 4 comprises six functional domains and remains the least understood, although its partial structure suggests unique roles different from those of the other isoforms. All isoforms contain the DWNN (17).

The structural diversity of RBBP6 isoforms allows them to engage in various cellular processes, including transcriptional regulation, mRNA splicing, and apoptotic signaling. The ability of RBBP6 isoforms to interact with various molecular components reflects their functional plasticity. Unless otherwise stated, the RBBP6-mediated cancer-promoting activities described in this review refer to the full-length, extensively characterized Isoform 1 (denoted RBBP6).

## 3 Expression pattern and mutations of RBBP6 in carcinogenesis

RBBP6 isoforms exhibit dysregulated expression across multiple cancers, with higher levels in advanced stages (10, 30). This dysregulation has been reported in lung (10), esophageal squamous cell (31), breast (9), cervical (16, 32), pancreatic (33), and other tumors (34). High levels of RBBP6 expression are associated with increased tumor proliferation, invasiveness, and resistance to therapy (9, 10, 34).

RBBP6 mutations identified by Harutyunyan et al. (35) were all located in the vicinity of its p53-binding domain, suggesting that they may affect p53 functions. The authors proposed that mutant RBBP6 could increase the rate of somatic mutagenesis by inhibiting p53 function and deregulating the cell cycle (35). RBBP6 has distinct mutational signatures in specific cancers, reflecting its multifaceted role in tumorigenesis. Germline mutations in RBBP6 are particularly common in familial myeloproliferative neoplasm (MPN) patients and predispose them to MPN development, occurring in 5% of familial and 0.6% of sporadic MPNs (35). Direct associations have been found between germline mutations in RBBP6 isoform 3 and susceptibility to MPN (36). Although familial MPN cases represent approximately 5% of all cases, specific mutations in RBBP6 isoform 3, including R1451T, R1569H, E1654G, A1673V, and S1444E, have been documented in familial MPN (35, 36). These mutations suggest a genetic predisposition to developing MPN, with RBBP6 potentially acting as an oncogene by promoting abnormal cell proliferation in hematopoietic tissues.

In another study, researchers genotyped single-nucleotide polymorphisms (SNPs) from the RBBP6 gene in a hospital-based case–controlled study of 992 cases and 1008 controls to investigate the link between SNPs in RBBP6 and gliomas in the Chinese Han population. Their data revealed that RBBP6 gene variants were significantly associated with glioma and increased risk (37), although no molecular mechanisms were proposed. The authors genotyped 10 SNPs and reported that four were significantly associated with glioma. Interestingly, different genetic variants are associated with various stages of disease. For example, rs2033214 was significantly associated with low-grade gliomas, and rs9933544 and rs13332653 were significantly associated with glioblastoma multiforme. However, rs11860248 was significantly associated with low-grade gliomas and glioblastomas (37). The aforementioned SNPs are all located within introns of the RBBP6 gene, as revealed by the Ensembl Variant Effect Predictor (38). Interestingly, these mutations do not directly alter the protein sequence and therefore specific domains of RBBP6. Nevertheless, studies have revealed that intronic mutations can still influence gene expression by disrupting splicing, creating pseudoexons, or altering regulatory elements, depending on their position and context (39). The limitation of this study is that other ethnic groups should be considered to ascertain the risk of RBBP6 mutations and glioma incidence.

## 4 RBBP6 as an E3 ubiquitin ligase and its associated roles in carcinogenesis

Ubiquitination is a highly conserved posttranslational modification process among eukaryotes that regulates fundamental biological processes such as protein turnover, cell cycle progression, immune mechanisms, autophagy, DNA damage responses, and homeostasis (40). The ubiquitin system is an enzymatic machinery that covalently modifies protein substrates with a 76-amino-acid residue protein, ubiquitin (40). E3 ligases, including RBBP6, regulate carcinogenesis via ubiquitin-mediated cellular processes (41).

**Figure 2:**
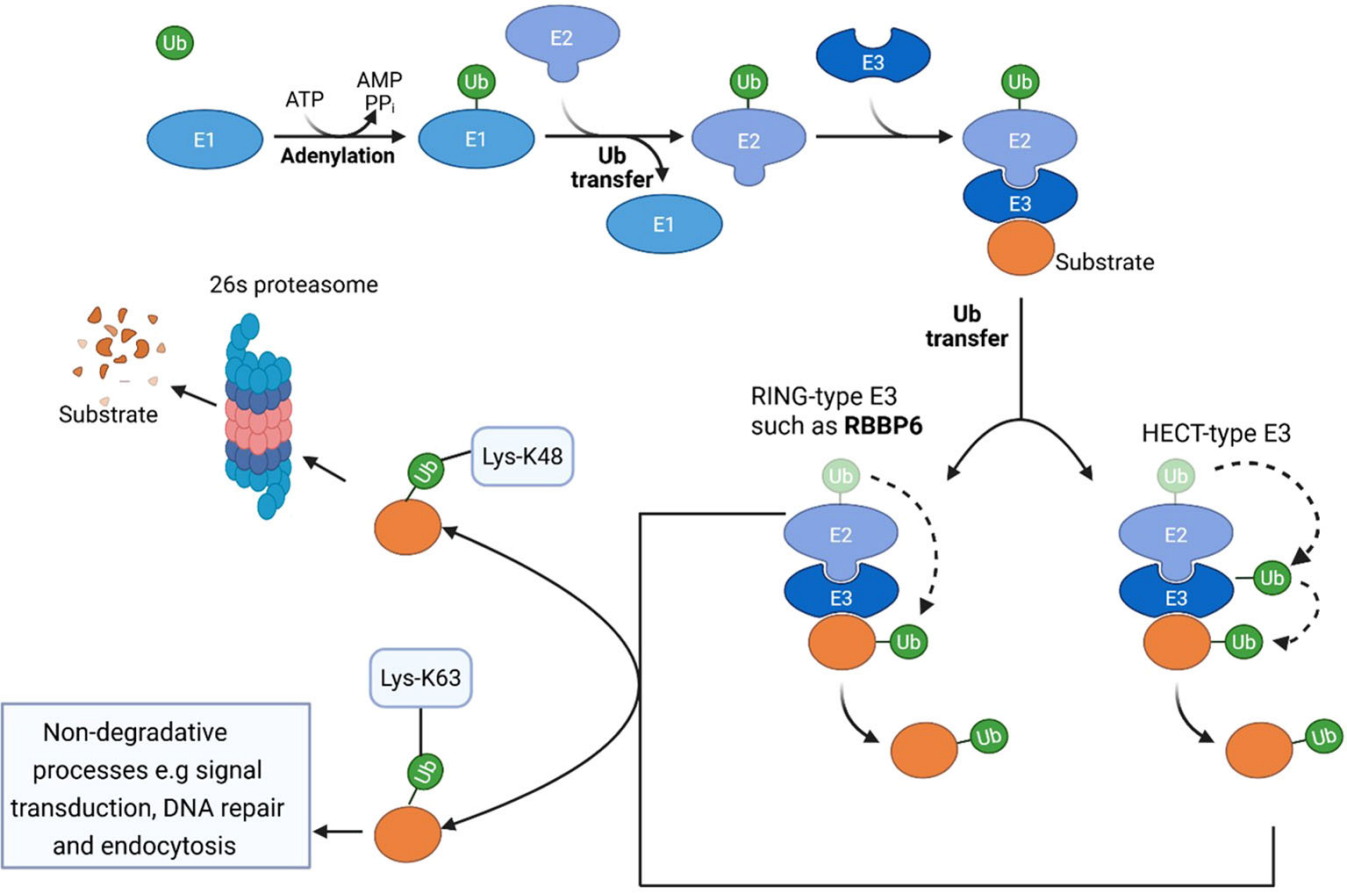
Ubiquitin–proteasome system. The first step in the ubiquitination cascade involves E1 enzymes, which activate ubiquitin via ATP-dependent adenylation and form a thioester bond with ubiquitin on a catalytic cysteine residue. E2 Ubiquitin-Conjugating Enzymes: Activated ubiquitin is transferred to E2 enzymes via a transthioesterification reaction. E2 enzymes serve as intermediates that facilitate ubiquitin transfer from E1 to E3 ligases. E3 Ubiquitin ligases: These enzymes mediate substrate specificity by recognizing and binding target proteins, enabling the final transfer of ubiquitin from the E2 enzyme to the substrate. E3 ligases vary in their structural domains, including RING, HECT, and RBR types, which determine their mechanism of ubiquitin transfer. While RING-type E3 ligases such as RBBP6 facilitate the transfer of ubiquitin directly from E2 to the protein substrate, HECT-type E3 ligases transfer ubiquitin sequentially from E2 to E3 and finally to the protein substrate. Specific ubiquitin modifications, such as Lysine-48, tag the substrate for degradation by the 26-S proteasome, whereas other modifications, such as Lysine K-63, may stabilize the protein for other nondegradative functions. This image was created using Biorender software.

In the ubiquitin–proteasome pathway, ubiquitin molecules are added to protein substrates by a three-enzyme ubiquitination cascade involving a ubiquitin-activating enzyme (E1), the ubiquitin-conjugating enzyme E2, and a ubiquitin ligase (E3), a process in which deubiquitinating enzymes can reverse (42, 43). E3 ubiquitin ligases recognize and covalently link specific protein substrates to ubiquitin molecules. Biological outcomes vary by ubiquitin conjugation type. For example, K48-linked polyubiquitination activates 26S proteasomal protein degradation (44), whereas K63-linked polyubiquitination controls nondegradative processes such as signal transduction, DNA repair, and endocytosis (45). **Figure 2** illustrates the ubiquitin system and highlights the role of RBBP6 as a RING-type E3 ubiquitin ligase.

Deregulation of the ubiquitin pathway has been reported in cancer (46). RBBP6 functions as an E3 ubiquitin ligase through its RING finger domain to promote carcinogenesis in several types of cancer, and several proteins have been reported to be ubiquitination substrates for RBBP6. Similarly, SNAMA, the *Drosophila* homolog of RBBP6, was also reported to exhibit E3 ubiquitin ligase activity through its RING finger domain (47). One of the first RBBP6 ubiquitination substrates discovered was Y-box-binding protein 1 (YB-1). Yang et al. (48) used the RBBP6 RING domain in a yeast two-hybrid screen to identify protein targets. They showed that RBBP6 suppresses YB-1, whose molecular functions include mRNA transcription and stability, splicing, DNA repair, translation, and oncogenesis. In addition, they demonstrated that RBBP6 uses its RING finger domain to polyubiquitinate YB-1 and tag it for proteasomal degradation (49). YB-1 stimulates proliferation in several cancers, such as breast cancer. While YB-1 is linked to cancer, its direct association with RBBP6 in carcinogenesis remains unknown (F. 48, 50, 51). Nevertheless, considering the involvement of YB-1 in carcinogenesis, it seems plausible that RBBP6 could influence carcinogenesis by ubiquitinating YB-1 and tagging it for proteasomal degradation.

RBBP6 also ubiquitinates several protein substrates associated with carcinogenesis, such as NF-kappa-B inhibitor alpha (IκBα), junction plakoglobin (JUP), and CPSF3, which are associated with the progression of colorectal cancer, thyroid cancer, and glioblastoma, respectively (3, 11; Z. 52). RBBP6 also ubiquitinates other substrates that are associated with carcinogenesis, including the zinc finger and BTB domain-containing 38 protein (ZBTB38) (4), growth factor receptor-bound protein 2 (GRB2) (53), and estrogen-related receptor alpha (ERRα) (54). Moreover, RBBP6, although not proven to ubiquitinate p53 directly, is known to act as a scaffold protein, thereby enhancing MDM2-mediated ubiquitination and proteasomal degradation of p53, with profound implications in carcinogenesis (55, 56). Details of the ubiquitination mechanisms of RBBP6 and outcomes are outlined in **Table 1**.

**Table 1:** RBBP6 and its ubiquitination substrates.

RBBP6 Substrate	Amino acid residue involved	Mechanisms and outcome	References
Y-box-binding protein 1 (YB-1)	Unknown	Polyubiquitination and proteasomal degradation of YB-1 lead to a decrease in the transactivational activity of YB-1.	(49)
Zinc Finger and BTB Domain Containing 38 (ZBTB38)	K112 and K804	Proteasomal degradation of transcriptional repressor ZBTB38, which suppresses transcription and levels of the MCM10 replication factor on chromatin, leading to increased replication and decreased DNA damage.	(4, 57)
NF-kappa-B inhibitor alpha (IκBα)	Unknown	Ubiquitination and proteasomal degradation of IκBα result in increased NF-κB-signaling and enhanced EMT and metastasis in colorectal cancer.	(11)
Growth factor receptor-bound protein 2 (GRB2)	K109	Polyubiquitination by E3 ubiquitin ligase RBBP6 releases MRE11, promoting homology-dependent DNA repair and genome stability.	(53)
Cleavage and polyadenylation specific factor subunit 3 (CPSF3)	K63	Polyubiquitination of CPSF3 thereby promotes its stability and leads to enhanced CPSF3-dependent alternative polyadenylation and cancer progression in glioblastoma.	(3)
Junction plakoglobin (JUP)	K48	The circular RNA, circSSU72, facilitates the binding of RBBP6 with the ARM domain of JUP protein, thereby inducing K48-ubiquitination of JUP by RBBP6, promoting glycolysis and metastasis of thyroid cancer.	(52)
Carbohydrate-responsive element-binding protein (ChREBP)	Unknown	circMlxipl promotes Rbbp6-mediated ubiquitination and proteasomal degradation of ChREBP in lipotoxic pancreatic β-cells.	(2)
Estrogen-related receptor alpha (ERRα)	K48 through the K100 residue	RBBP6-mediated polyubiquitination and subsequent proteasomal degradation of ERRα promote mitochondrial dysfunction in renal tubular cells in diabetic renal disease.	(54)
Forkhead box P3 (FOXP3)	K48	Ubiquitination of FOXP3 promotes glucose metabolism, cell proliferation, migration, invasion, and angiogenesis in HBV-related hepatocellular carcinoma.	(58)

In a recent study, CRISPR/Cas9 knockout screens targeting human deubiquitinases and E3 ubiquitin ligases revealed that RBBP6 plays a critical role in maintaining glioblastoma stem cells (GSCs). GSCs drive tumor initiation, angiogenesis, and therapy resistance. (59). shRNA-mediated knockdown of RBBP6 inhibited the proliferation of glioblastoma stem cells and inhibited tumor initiation (3). RBBP6 mediates K63 ubiquitination and stabilization of cleavage and polyadenylation specific factor 3 (CPSF3). Stabilized CPSF3 promoted alternative polyadenylation events, which upregulated MYC expression in glioblastoma stem cells. Moreover, pharmacological inhibition of CPSF3 phenocopies RBBP6 knockdown by suppressing tumor growth *in vivo* (3).

## 5 Roles of RBBP6 in DNA replication and repair

Genome integrity depends on faithful DNA replication and robust repair mechanisms. Aberrant DNA replication over multiple cell divisions can trigger sufficient mutations that initiate driver mutations and initiate carcinogenesis (60). RBBP6 plays a critical role in maintaining genome integrity by controlling DNA replication proteins. RBBP6 regulates DNA replication and chromosomal stability at common fragile sites by interacting with ZBTB38 and MCM10 (4). These authors reported that RBBP6 ubiquitinates and destabilizes the transcriptional repressor protein ZBTB38, suppressing the expression of MCM10, which is vital in DNA replication. RBBP6 knockdown suppresses replication fork movement. Simultaneous knockdown of RBBP6 and ZBTB38 rescued cells from DNA damage, suggesting that the resulting DNA damage from RBBP6 depletion depends on ZBTB38. Western blots revealed that RBBP6-depleted cells accumulate the following DNA damage markers: γH2AX, phosphorylated ATM, and Chk2 (4).

Another study reported that endogenous RBBP6 is localized at DNA damage sites, which are characterized by γH2AX. By employing UV laser microirradiation-coupled immunofluorescence, researchers demonstrated that RBBP6 polyubiquitinated a novel substrate, GRB2, which colocalized with RBBP6 at DNA damage sites in the nucleus (53). Mechanistically, they showed that RBBP6-mediated K109 polyubiquitination of GRB2 at DNA damage sites released meiotic recombination 11 homolog A (MRE11), which promoted homology-directed DNA repair (HDR) (53). Conversely, they reported that RBBP6 depletion led to a prolonged GRB2-MRE11 complex and dysfunctional HDR (53).

## 6 RBBP6 promotes resistance to chemotherapy and radiotherapy

Chemotherapy and radiotherapy impair replication and DNA integrity, leading to cell cycle arrest and cell death. Chemotherapy often targets rapidly dividing cells by interfering with DNA replication or division, whereas radiation therapy directly damages DNA, hindering replication and cell division (61). Cellular responses to DNA damage are important determinants of cancer development and outcome following radiation and chemotherapy. RBBP6 promotes cancer cell resistance to radiotherapy and chemotherapy, and RNAi-mediated depletion of RBBP6 reportedly improves the sensitivity of RBBP6 to chemotherapy and radiotherapy (12, 31, 32).

### 6.1 RBBP6 enhances radioresistance

Radiotherapy induces DNA damage directly by ionizing radiation or indirectly via free radicals (62). RBBP6 contributes to radioresistance in several types of cancer. For example, RBBP6 knockdown increases radiosensitivity in CRC cells (31), suggesting RBBP6 is a therapeutic target for preoperative radiotherapy. RBBP6 contributes to gastric cancer progression and is an independent prognostic biomarker in this disease. In gastric cancer cells, silencing RBBP6 increases cellular sensitivity to radiotherapy in a p21- and p53-dependent manner (63). p21 induces G2/M arrest, the cell cycle phase most vulnerable to radiotherapy (64, 65).

### 6.2 RBBP6 enhances chemotherapy resistance

Studies show that RBBP6 expression influences chemoresistance in cancer cells. Knockdown of RBBP6 sensitizes cells to chemotherapy. For example, siRNA-mediated knockdown of RBBP6 sensitizes cervical cancer cells to cisplatin-mediated apoptosis by suppressing B-cell lymphoma 2 (BCL-2) expression, and knockdown of RBBP6 renders breast cancer cells susceptible to staurosporine- and camptothecin-mediated cell death (9, 32). Moreover, high expression of RBBP6 was observed in ovarian cancer cell lines. Silencing RBBP6 reversed cisplatin resistance in p53-positive cells but not in p53-mutant cells, implicating the p53 pathway in enhanced sensitivity to chemotherapy (12). RBBP6 upregulation enhances cisplatin resistance through BCL-2 suppression, and silencing RBBP6 could contribute to chemotherapeutic efficacy and reduce resistance to cisplatin treatment in cervical cancer (32). Moreover, another study revealed that RBBP6 expression was significantly higher in cisplatin-resistant ovarian cancer cells than in cisplatin-sensitive ovarian cancer cells and that the knockdown of RBBP6 sensitized cells to cisplatin-mediated apoptosis in a p53-dependent manner (12).

## 7 Interplay of RBBP6 and p53 in cancer

p53, the ‘guardian of the genome,’ orchestrates DNA repair and genomic stability. Considerable efforts have been invested in studying the p53 pathway, which is known to be defective in almost all cancers and mutated or inactivated in over 50% of cancers. Under normal cellular conditions, p53 is expressed at low levels. However, its expression is increased following radiation-mediated DNA damage. In response to varying levels of DNA damage, p53 regulates cell cycle arrest, apoptosis, and senescence, responses that are crucial in deciding cell fate (66).

RBBP6 does not directly ubiquitinate p53 but facilitates HDM2-mediated p53 degradation (55). p53 regulates apoptosis partly by inducing the expression or activation of the proapoptotic protein BCL-2-associated X protein (Bax) through transcription-dependent and transcription-independent mechanisms (67, 68). Bax activation promotes mitochondrial membrane permeabilization, leading to caspase-3 activation and eventual apoptosis (69). p53 also harnesses transcription-dependent and transcription-independent pathways to suppress the expression of the prosurvival gene *BCL-2* (70). The ability of RBBP6 to negatively regulate p53 has profound implications in promoting tumorigenesis in several types of cancers, including colorectal, lung, breast, and ovarian cancers, as exemplified below.

### 7.1 Colorectal cancer

Approximately 50% of all tumors and 75% of all invasive colorectal cancers harbor p53 mutations. p53 mutation or loss can compromise cellular sensitivity to chemotherapy and radiotherapy, leading to therapeutic resistance and worse clinical outcomes, with increasing malignancy associated with a greater mutational frequency of p53 (71). Chen et al. (72) investigated the relationship and significance of mutant TP53 and RBBP6 overexpression in colon cancer progression. An analysis of samples from 203 patients with CRC revealed that the overexpression of RBBP6, combined with or without mutant TP53, predicts a poor prognosis in patients with CRC.

In colon cancer, RBBP6 expression is correlated with tumor grade, metastasis, stage, and invasion depth (72). Moreover, the presence of mutant TP53 and RBBP6 overexpression was associated with poor prognosis in colon cancer patients. This study further demonstrated that patients with RBBP6-overexpressing and mutant TP53-overexpressing tumors relapsed and died within a significantly shorter period after surgery than those with RBBP6-overexpressing tumors alone. Moreover, the expression of RBBP6 independently predicts disease-free survival and overall survival. This study suggests that RBBP6 has prognostic significance and could be a therapeutic target in colon cancer.

### 7.2 Lung cancer

Recent studies have identified the critical role of RBBP6 in the development and progression of lung cancer. Its frequent overexpression in lung cancer contributes to tumorigenesis, primarily through interactions with key tumor suppressor proteins, including p53 and pRb (10a). RBBP6 knockdown increased the expression of the proapoptotic protein, Bax, and decreased the expression of the antiapoptotic protein, BCL-2, in lung cancer cells, suggesting that RBBP6 may have an antiapoptotic effect on lung cancer cells by regulating the Bax/BCL-2 ratio (10). In addition, functional studies have indicated that silencing RBBP6 significantly inhibits the proliferation of A549 cells and suppresses tumor growth in xenograft models, suggesting its critical role in promoting tumor growth (73). Clinical data further corroborate this oncogenic function, as NSCLC patients with elevated RBBP6 expression exhibit significantly shorter overall survival than patients with low RBBP6 expression (73). These findings indicate that RBBP6 might have value as a prognostic biomarker and a therapeutic target for NSCLC patients, particularly with respect to cancer progression.

### 7.3 Breast cancer

Breast cancer progression involves complex genetic and molecular mechanisms characterized by hallmark features such as the ability to evade growth suppressors, resist apoptosis, sustain proliferative signaling, and acquire replicative immortality (74). Breast cancer subtypes present different p53 expression profiles, ranging from wild-type to mutated forms (75), affecting tumor behavior and therapeutic responses. Elevated levels of RBBP6 in breast cancer are associated with increased tumor aggression, poor differentiation, and resistance to apoptosis (9). One study demonstrated that silencing RBBP6 expression significantly inhibits procancerous pathways in breast cancer cell lines irrespective of their p53 status (9), suggesting its potential as a therapeutic target in breast cancer. Another study reported that MCF7 breast cancer cells overexpress RBBP6 isoform 1 but lack isoforms 2 and 3. Treating MCF7 cells with the FDA-approved chemotherapeutic drug arsenic trioxide induced G2/M cell cycle arrest, upregulated Bax protein expression, and induced caspase-dependent apoptosis. Moreover, curcumin treatment of MCF7 cells suppressed RBBP6 expression and stimulated apoptosis in these cells (76).

### 7.4 Ovarian cancer

Ovarian cancer is the deadliest gynecologic malignancy (77). RBBP6 overexpression in ovarian cancer has been associated with poor clinical outcomes. Functional studies by Liu et al. (78) demonstrated a significant correlation between RBBP6 expression and lymphatic and distant metastasis. Silencing RBBP6 in ovarian cancer cells suppressed proliferation and cell migration *in vitro* and significantly slowed tumor growth *in vivo* (78). At the molecular level, RBBP6 promotes its oncogenic functions by upregulating phosphoinositide-3-kinase regulatory subunit 6 (PIK3R6) (78), a target gene that rescues proliferation and migration in cells that lack RBBP6. The decreased proliferative and migratory potentials in ovarian cancer cells with RBBP6 knockdown were abolished by overexpressing PIK3R6. Furthermore, RBBP6 knockdown delayed the *in vivo* growth of ovarian cancer in nude mice, while PIK3R6 overexpression restored the slower cancer progression (78).

RBBP6 and p53 are known to contribute to resistance to cisplatin chemotherapy in ovarian cancer. Researchers reported that RBBP6 expression is elevated in ovarian cancer, and when RBBP6 was silenced in wild-type p53 A2780 ovarian cancer cells, significant cell death was observed, unlike in p53-mutant SKOV3 cells. RBBP6 knockdown significantly elevated cisplatin-induced p53, Bax and p21 while decreasing BCL-2 expression (12). To determine the role of p53, they demonstrated that inhibiting p53 remarkably reversed the effect of RBBP6 knockdown on increased cell sensitivity to cisplatin in A2780 cells by increasing cell viability and suppressing apoptosis (12).

## 8 Noncoding RNAs regulate RBBP6 in cancer progression

Recent studies have reported that noncoding RNAs such as long noncoding RNAs (lncRNAs), microRNAs (miRNAs), and circular RNAs (circRNAs) interact with RBBP6 to influence cancer progression (2, 8, 13, 14). miRNAs are short, single-stranded RNAs (21–25 nucleotides) that regulate gene expression and have been implicated in carcinogenesis (14, 52, 79–81). LncRNAs, which critically regulate cancer-associated processes, usually contain several miRNA-binding sites, which enable them to act as miRNA sponges, thereby regulating gene expression (82). lncRNAs bind miRNAs at complementary sites, thereby preventing them from binding to their target mRNAs (80, 82). circRNAs are a large group of noncoding RNAs with a covalent loop structure devoid of 5′-cap and 3′-poly (A) structures, making them highly resistant to exonuclease degradation (83, 84). circRNAs also act as competitive endogenous RNAs (ceRNAs), which can ‘sponge’ miRNAs to regulate mRNA expression (84). circRNAs are known to participate in cancer initiation, progression, and therapeutic response (79, 84). Noncoding RNAs regulate RBBP6 activity and expression in several types of cancer, thereby influencing cancer progression. The roles of noncoding RNAs and transcription factors in RBBP6-mediated cancer progression are highlighted in **Table 2**.

**Table 2:** Regulation of RBBP6 in carcinogenesis by noncoding RNAs and transcription factors.

Regulator/molecule type	Regulatory mechanism	Cell type	References
Noncoding RNAs			
miR−193a−5p	Downregulated miR-193a-5p expression may promote RBBP6-mediated tumor promotion.	Epithelial ovarian cancer	(81)
LncRNA SLCO4A1/miR-876-3p	LncRNA SLCO4A1 interacts with miR876-3p, thereby upregulating RBBP6, which activates JNK signaling to drive proliferation.	Acute lymphoblastic leukemia	(8)
miR-590-3p	RBBP6 knockdown stimulated the release of miR-590-3p from shortened UTRs, thereby decreasing MYC expression.	Glioblastoma stem cells	(3)
lncRNA FGD5-AS1/miR-107	FGD5-AS1 targets miR-107 to promote RBBP6 expression, thereby aggravating ovarian cancer progression	SKOV3 ovarian cancer cell line	(85)
miR424	miR424 interacts with the 3’-UTR of RBBP6, suppressing its mRNA expression.	Cervical cancer cell lines	(86)
circSSU72	circSSU72 enhances binding between RBBP6 and JUP, thereby mediating K48-specific ubiquitination of JUP by RBBP6, thus promoting thyroid cancer glycolysis and metastasis by activating the beta-catenin pathway.	Thyroid cancer cell lines	(52)
Transcription factors			
ELF-1 and EGR1/Transcription factors	ELF1 and EGR1 transcription factors are bound to the hypomethylated promoter region of RBBP6, thereby upregulating RBBP6 expression.	Colorectal cancer	(11)
Regulatory factor X5 (RFX5)	RFX5 increases RBBP6 transcription by binding its promoter in HBV-positive HCC cells.		(58)

### 8.1 Noncoding RNAs and RBBP6 in ovarian cancer

miRNAs have been identified as key factors in the pathogenesis of ovarian cancer, with miR-193a-5p emerging as an important regulator, although its mechanism of action remains incompletely defined. Recent studies revealed a significant interaction between miR-193a-5p and RBBP6, where downregulated miR-193a-5p in epithelial ovarian cancer (EOC) correlated with elevated RBBP6 expression (81). Increased RBBP6 levels drive tumorigenic processes, including increased proliferation, migration, and invasion, while suppressing apoptosis. Restoration of miR-193a-5p suppresses these oncogenic activities, whereas RBBP6 overexpression reverses these effects, which promotes miR-193a-5p as a tumor suppressor and RBBP6 as an oncogenic driver in EOC (81).

The lncRNA FGD5-AS1 is involved in the malignant progression of several types of cancer, including ovarian cancer, and is also known to regulate miRNAs such as miR-142-5p and miR-107 to promote ovarian cancer progression (14, 85). One study employed *in silico* methods to predict that FGD5-AS1 contains binding sites for miR-107. Researchers further established that the FGD5-AS1/miR-107/RBBP6 axis could be involved in ovarian carcinogenesis (85). Mechanistically, FGD5-AS1 was shown to target miR-107 to promote the expression of RBBP6, thereby aggravating ovarian cancer progression (85). Knockdown of RBBP6 or FGD5-AS1 inhibited angiogenesis and cell proliferation, whereas their overexpression promoted these processes (85). Similarly, another study used an *in silico* tool, TargetScan, to predict that RBBP6 was a target gene of miR-193a-5p. Serum analysis confirmed inverse expression patterns between RBBP6 and miR-193a-5p (81). RBBP6 expression was significantly upregulated, whereas miR-193a-5p was downregulated in the sera of ovarian cancer patients compared with healthy subjects. They further demonstrated that RBBP6 was the target gene for miR−193a−5p. To support their prediction, they revealed that a miR−193a−5p mimic significantly stimulated apoptosis and repressed the proliferation, migration, and invasion of SKOV3 ovarian cancer cells (81).

### 8.2 Noncoding RNAs and RBBP6 in cervical cancer

Several miRNAs play pivotal roles in cervical cancer progression and are suggested to be biomarkers for this disease (87). A typical example is miR424, a tumor suppressor whose expression is inversely correlated with promoter DNA methylation in some cancers and whose suppression is associated with cervical cancer progression (86). RBBP6 was reported to be a direct target of miR-424. Mechanistically, miR424 interacts with the 3’-UTR of *RBBP6*, suppressing its mRNA expression; however, DNA hypermethylation of the miR-424 promoter inhibits its expression, which may increase the expression of *RBBP6* (86). Hence, aberrant promoter methylation can disrupt miRNA activity, leading to its transcriptional silencing during cervical cancer progression. Targeting aberrant DNA methylation or dysregulated miRNA expression could suppress RBBP6 overexpression and ovarian cancer progression.

### 8.3 Noncoding RNAs and RBBP6 in leukemia and myeloproliferative neoplasia

Long noncoding RNAs are known to control the progression of several types of cancer, including acute lymphocytic leukemia (ALL). Although studies are limited, RBBP6 likely contributes to leukemia and myeloproliferative neoplasms (MPNs) by regulating the cell cycle and apoptosis. ALL is a hematologic cancer induced by the proliferation of immature T-lymphocyte clones. The upregulation of RBBP6 has been associated with the growth of ALL cells, suggesting an oncogenic role (8). The underlying mechanism involves the regulation of the lncRNA SLCO4A1 antisense RNA 1 (SLCO4A1-AS1), which competes for miR-876-3p and promotes ALL cell proliferation through the JNK signaling pathway by increasing the expression of RBBP6. Furthermore, the upregulation of RBBP6 expression was shown to counteract the inhibitory effect of SLCO4A1-AS1 knockdown, supporting the role of RBBP6 in improving cell proliferation and potentially contributing to the oncogenic progression of ALL (8). The oncogenic function of RBBP6 in this context is highlighted by its participation in cell growth and survival, possibly by modulating critical signaling pathways associated with cell division and resistance to apoptosis.

### 8.4 Noncoding RNAs and RBBP6 in thyroid cancer

RBBP6 has recently been reported to interact with the circular RNAs circSSU72 and circMlxipl to promote carcinogenesis and type 2 diabetes, respectively (2, 13, 52). One study reported that circSSU72 promotes thyroid cancer by functioning as a sponge for miR-451a to influence cell proliferation, migration, and invasion in thyroid cancer (13). Moreover, a recent study revealed that circSSU72 enhances the binding between RBBP6 and JUP, thereby mediating K48-specific ubiquitination of JUP by RBBP6. JUP ubiquitination promotes thyroid cancer glycolysis and metastasis by activating the beta-catenin pathway (52). The authors showed that RBBP6-induced ubiquitination of JUP was rescued by silencing circSSU72.

## 9 Role of RBBP6 in immunoregulation

Cancer immunotherapy harnesses the body’s inherent anticancer ability to target and destroy tumor cells using humoral and T-cell-based strategies. RBBP6 is a promising target for cancer immunotherapy, as its overexpression strongly correlates with cancer progression in several types of cancer (10, 11, 34, 88). Cytotoxic T cells targeting RBBP6-derived peptides successfully destroy esophageal cancer cells *in vitro* and trigger tumor shrinkage in mouse xenograft models (88). Elevated levels of RBBP6 expression have been reported in esophageal cancer cells, with overexpression associated with enhanced cell growth and reduced patient survival rates (88). Localization studies further support the oncogenic role of RBBP6 in esophageal cancer, as high expression of RBBP6 transcript 3 was found in esophageal tissues, and the mRNA of the RBBP6 transcript 3 was also localized in the normal tissue surrounding the tumor (17). These findings suggest that RBBP6 may influence both tumorigenesis and the tumor microenvironment.

Yoshitake et al. (88) reported markedly upregulated expression of *RBBP6* mRNA and protein in esophageal cancer tissues and cell lines, which correlated with poor prognosis in postsurgical patients who presented with microscopic residual tumor cells (88). They revealed using immunohistochemistry that, compared with normal esophageal epithelial cells, most esophageal cancer tissues (20 out of 22) presented significantly elevated expression of the RBBP6 protein. These findings suggest that RBBP6 is instrumental in the pathogenesis of esophageal cancer. These authors successfully generated cytotoxic T lymphocytes (CTLs) from HLA-A24–positive esophageal cancer patients using RBBP6-specific peptides and demonstrated that these CTLs induced cytotoxicity in RBBP6- and HLA-A24-positive cancer cell lines. Moreover, the RBBP6–specific CTL line suppressed the tumor growth of a human esophageal cancer cell line engrafted in nude mice. These data suggest that RBBP6 could function as a tumor antigen for immunotherapy in esophageal cancer patients (88).

## 10 RBBP6 interacts with signaling pathways to promote cancer progression

Aberrant cell signaling plays a crucial role in carcinogenesis. Compelling evidence indicates that RBBP6 promotes carcinogenesis by activating the JNK and NF-κB signaling pathways, which are crucial for cell viability, proliferation, and metastasis (8, 11, 16).

### 10.1 RBBP6 and NF-κB signaling

NF-κB is a key transcription factor that controls the expression of essential genes that regulate immunity, inflammation, apoptosis, and cell proliferation. NF-κB activity is associated with cancer initiation, progression, and metastasis (89, 90). RBBP6 promotes NF-κB translocation to the nucleus, thereby inducing epithelial-to-mesenchymal transition by increasing the expression of mesenchymal cell markers (Vimentin, N-cadherin, and Snail) and decreasing the expression of epithelial cell markers (E-cadherin) in CRC, thereby promoting invasion and metastasis (11). The authors showed that RBBP6 is an independent prognostic biomarker in CRC and proved that RBBP6 expression was related to histological grade and TNM stage. RBBP6 was identified as a prognostic factor and predicted a poor outcome for CRC patients. RBBP6 promoted cell proliferation, migration, and invasion in CRC cells and promoted tumor growth, lung metastasis, and liver metastasis in mouse models (11).

**Figure 3:**
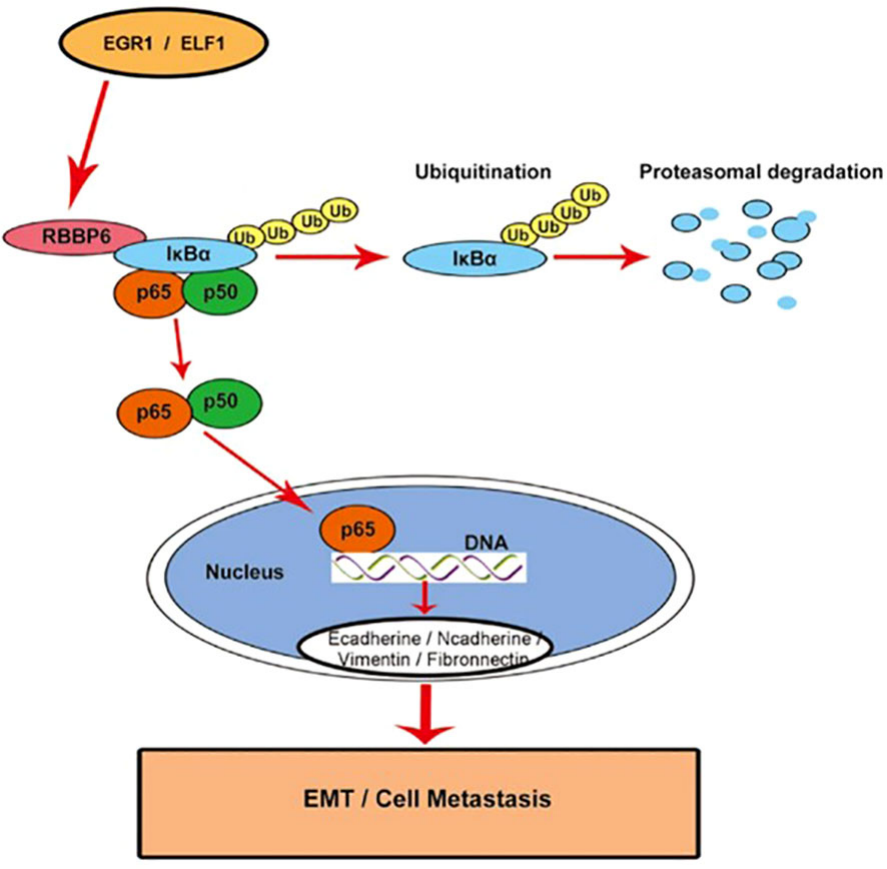
Model showing the interplay between RBBP6 and NF-κB during EMT in colorectal cancer. RBBP6 expression is activated by oncogenic transcription factors such as ELF1 and EGR1. RBBP6 ubiquitinates IkBα, which releases the NF-κB (p65/p50) complex to transactivate mesenchymal cell markers such as vimentin, N-cadherin, and Snail and suppresses the expression of epithelial cell markers such as E-cadherin, thereby promoting metastasis in colorectal cancer. Image reproduced from Xiao et al. (11), licensed under CC BY 4.0.

Mechanistically, RBBP6 bound to and ubiquitinated the NF-κB inhibitor IκBα, which stimulated NF-κB activation and then induced epithelial-to-mesenchymal transition and metastasis (**Figure 3**). To determine the cause of RBBP6 overexpression in colorectal cancer, they used DNA methylation and ChIP analysis, which revealed that the promoter of RBBP6 was hypomethylated and activated by several oncogenic transcription factors, such as EGR1 and ELF1. This pioneering study is one of the first to report the mechanism involved in RBBP6 overexpression in cancer by associating RBBP6 promoter hypomethylation and activation by oncogenic transcription factors, which leads to the upregulation of its expression in colorectal cancer (**Figure 3**). These data suggest that RBBP6 may be a potential prognostic biomarker and therapeutic target for CRC invasion and metastasis (11).

### 10.2 RBBP6 and JNK signaling

#### 10.2.1 Cervical cancer

**Figure 4:**
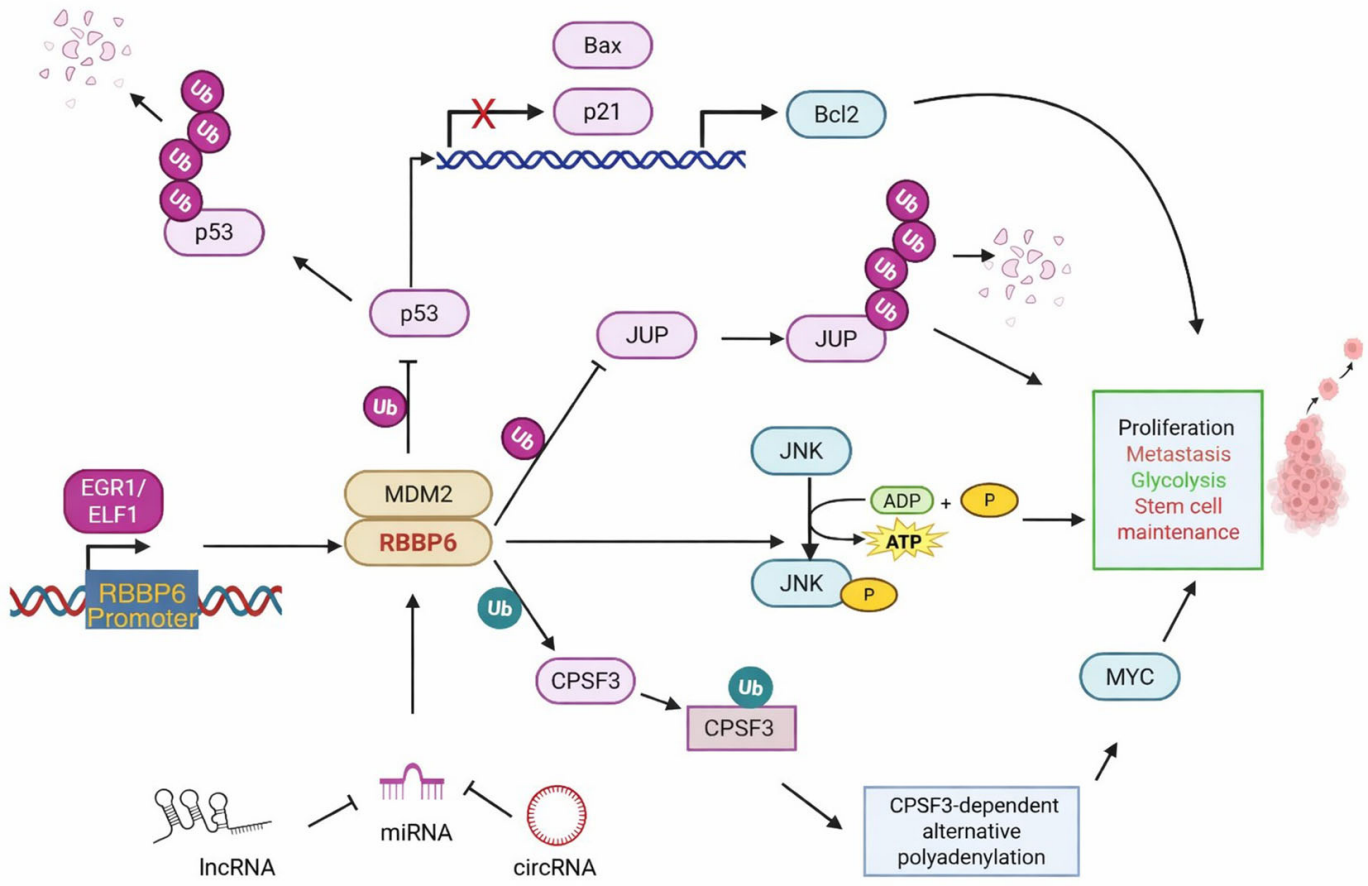
Multifaceted mechanisms of RBBP6 in cancer progression. RRRBP employs multiple strategies to promote cancer, primarily through its principal function as an E3 ubiquitin ligase. The promoter region of RBBP6 is hypomethylated and bound by multiple oncogenic transcription factors, such as EGR1 and ELF1, which activate RBBP6 expression in colorectal carcinogenesis. RBBP6 facilitates MDM2-mediated ubiquitination and subsequent proteasomal degradation of p53, thereby suppressing Bax and p21 expression and promoting BCL-2 expression, leading to proliferation and metastasis. RBBP6 also mediates JUP ubiquitination, which activates glycolysis and stimulates cell proliferation in thyroid cancer. RBBP6 K63-linked ubiquitination of CPSF3 stabilizes CPSF3 to regulate alternative polyadenylation events, thereby upregulating MYC expression and maintaining glioblastoma stem cells. RBBP6 stimulates the phosphorylation of JNK, activating cell proliferation and metastasis in cervical and prostate cancers. Noncoding RNAs such as circular RNAs, lncRNAs, and miRNAs influence RBBP6-mediated ubiquitination of its substrates. Green Ub indicates protein stabilization following ubiquitination while pink Ub refers to ubiquitination that leads to proteasomal degradation. This image was created using Biorender software.

RBBP6 displays multiple mechanisms in cancer progression, including interactions with JNKs (**Figure 4**). JNKs are a highly conserved group of mitogen-activated protein kinases involved in various cellular processes, including cell survival, death, and inflammation, and significantly influence cancer initiation, progression, and response to therapy (91). RBBP6 expression is correlated with c-Jun N-terminal kinase (JNK1/2) signaling in cervical cancer. RBBP6 overexpression in cervical squamous carcinoma tissues was reported to increase the phosphorylation of c-Jun NH2-terminal kinase (p-JNK), which in turn promoted cell viability and migration in SiHa and C33a cell lines (16b). Compared with the control, the overexpression of RBBP6 in cervical cancer cell lines induced increased levels of phosphorylated JNK, which led to increased cell viability, proliferation, and migration (16b). To investigate the effect of JNK on the observed phenotype, the authors showed that stimulating the cells with a JNK-specific inhibitor abrogated RBBP6-mediated proliferation. Furthermore, silencing RBBP6 in CaSki cells was found to reduce telomerase activity by downregulating hTERT, which would counteract the cellular immortality typically observed in cancer cells (92). In contrast, as a tumor suppressor, RBBP6 may also exert its effects by promoting apoptosis. For example, RBBP6 expression was determined to be particularly high at sites of increased apoptosis in cervical carcinoma tissues, suggesting its involvement in the regulation of apoptosis (34).

Therefore, the capacity of RBBP6 to function as both an oncogene and a tumor suppressor in cervical cancer depends on its expression levels and the cellular context, influencing key pathways such as apoptosis, cell proliferation, and telomere maintenance. These findings underscore the complexity of the role of RBBP6 in cervical cancer and its potential as both a therapeutic target and a biomarker for prognosis and treatment response. **Figure 4** shows the diverse mechanisms by which RBBP6 is involved in cancer progression.

#### 10.2.2 Prostate cancer

Prostate cancer is the most common cancer in men and is generally characterized by slow growth and high survival rates. RBBP6 is overexpressed in prostate cancer, and its expression is correlated with poor prognosis in prostate cancer patients. Moreover, researchers have shown that RBBP6 expression is also correlated with tumor stage and grade, lymph node metastasis, and preoperative prostate‐specific antigen levels (15). Loss of RBBP6 suppressed cell proliferation, induced G2/M cell cycle arrest, and stimulated apoptosis. Interestingly, RBBP6 knockdown also suppressed the JNK pathway by inhibiting phosphorylated JNK in prostate cancer cell lines and decreased invasion and metastasis by inhibiting the matrix metalloproteinases MMP-2 and MMP-9 (15). In prostate cancer cells, western blot analysis showed that RBBP6 knockdown increased the expression of p53, pRb, Bax, and cleaved caspase-3 but decreased BCL-2 expression (15). Similarly, Motadi et al. (93) reported that RBBP6 knockdown enhanced p53 and Bax expression at both the mRNA and protein levels, increased caspase-3/7 activity *in vitro*, and sensitized prostate cancer mouse xenograft tumors to cannabidiol and cisplatin treatment.

#### 10.2.3 Acute lymphocytic leukemia

An intriguing molecular pathway involving lncRNAs, miRNAs, and RBBP6 was reported to activate JNK signaling and promote cell proliferation in acute lymphocytic leukemia cells (8). The long noncoding RNA SLCO4A1-AS1 can act as a sponge, binding to miR-876-3p and thereby enhancing *RBBP6* expression. Interestingly, SLCO4A1-AS1 activated the JNK signaling pathway by upregulating RBBP6. Overexpression of RBBP6 or activation of JNK signaling reversed the inhibitory effects of silencing SLCO4A1-AS1 on the proliferation of ALL cells (8).

The various roles of RBBP6 in distinct types of cancer are illustrated in **Table 3**.

**Table 3:** Role of RBBP6 in distinct types of cancer.

Cancer type	RBBP6 expression in cell line/tissue sample/model	Mechanism of RBBP6-mediated cancer progression	References
Acute lymphocytic leukemia (ALL)	Overexpressed in ALL cell lines	The lncRNA SLCO4A1-AS1/miR-876-3p/RBBP6 axis regulates cell proliferation and apoptosis in acute lymphocytic leukemia through the JNK signaling pathway.	(8)
Myeloproliferative neoplasms (MPN)	Germline mutations in Isoform 3 in patient-derived clinical samples with MPN	E1654G, R1451T, R1569H, S1444E and A1673V mutations of RBBP6 have been strongly associated with familial predisposition to MPN	(36)
Glioma and glioblastoma	Genetic variants of RBBP6 are significantly associated with glioma, and increased risk in the Chinese Han population	rs2033214 SNP is significantly associated with low-grade gliomas, and rs9933544 and rs13332653 SNPs are significantly associated with glioblastoma multiforme. The rs11860248 SNP is markedly associated with low-grade gliomas and glioblastomas.	(37)
Esophageal cancer	Overexpression in esophageal cancer cell lines and clinical tissue samples	The upregulation of RBBP6 was correlated with increased proliferation in an esophageal cancer cell line and low survival rates in patients with esophageal cancer.	(1)
Esophageal cancer	Transcript 3 is expressed in esophageal tissues	Localization of RBBP6 transcript 3 mRNA in the normal tissue around the tumor	(17)
Cervical cancer	Overexpressed in HeLa cell lines	RBBP6 expression promotes cisplatin sensitivity in HeLa cells through downregulation of BCL-2	(32)
Cervical cancer	Overexpressed in biopsy samples of cervical carcinoma	RBBP6 overexpression at sites with elevated levels of apoptosis	(34)
Cervical cancer	Overexpressed in specimens of human cervical squamous carcinoma. tissues	Overexpression of RBBP6 increases phosphorylated c-Jun NH2-terminal kinase (p-JNK) and proliferation in SiHa and C33a cell lines.	(16)
Hepatocellular carcinoma	Transcripts 1 and 3 are expressed in hepatocellular carcinoma tissues	Localization of RBBP6 transcript 3 mRNA in normal tissue around the tumor	(17)
Hepatocellular carcinoma	Overexpressed in HepG2 hepatocellular carcinoma cell line	High expression of RBBP6 transcript 3 and low expression of RBBP6 transcript 1 were observed in HepG2 cells.	(18)
Glioblastoma	mRNA expression of RBBP6 is upregulated in glioblastoma tissue compared to normal brain tissue	RBBP6 induces K63-linked polyubiquitination of CPSF3, promoting its stability and enhancing CPSF3-dependent alternative polyadenylation and cancer progression in glioblastoma. RBBP6 supports high MYC expression in glioblastoma stem cells by regulating CPSF3-dependent alternative polyadenylation. RBBP6 knockdown inhibited glioblastoma stem cell proliferation and tumor initiation.	(3)
Colorectal cancer	Overexpression is associated with histological grade, TNM stage, and metastasis in CRC patients.	RBBP6-mediated ubiquitination of IκBα promotes NF-κB-signaling and enhances EMT and metastasis in colorectal cancer. RBBP6 also increases radioresistance in colorectal cancer, and RBBP6 knockdown induces G2/M cell cycle arrest and sensitizes CRC cells to radiotherapy.	(11)
Lung cancer	Overexpressed in lung cancer	Knockdown of RBBP6 increased the expression of pro-apoptotic protein, Bax, and decreased anti-apoptotic protein, BCL-2, in lung cancer cells, suggesting that RBBP6 may have an anti-apoptotic function in lung cancer cells through regulation of the Bax/BCL-2 ratio.	(10)
Breast cancer	Overexpression in breast cancer is associated with increased tumor aggression, poor differentiation, and resistance to apoptosis.	RBBP6 aggravates p53 inactivation by promoting ubiquitination and degradation of p53 by the ubiquitin–proteasome pathway, thus inhibiting its tumor-suppressive functions in breast cancer.	(94) (9)
Ovarian cancer	Highly expressed in ovarian cancer	FGD5-AS1 was shown to target miR-107 to promote the expression of RBBP6, thereby aggravating ovarian cancer progression. RBBP6 or FGD5-AS1 knockdown inhibited angiogenesis and cell proliferation, while their overexpression promoted them.	(85)
Thyroid cancer	Expressed in thyroid cancer	circSSU72 binds to the armadillo domain of junction plakoglobin (JUP), promoting RBBP6-mediated K48-specific ubiquitination of JUP by enhancing the binding between JUP and RBBP6, thereby stimulating glycolysis and metastasis in thyroid cancer.	(52)
Prostate cancer	Overexpressed in prostate cancer, and its expression is correlated with poor prognosis	RBBP6 knockdown suppressed the JNK pathway by inhibiting phosphorylated JNK expression. RBBP6 induced G2/M cell cycle arrest and enhanced invasion and metastasis by upregulating matrix metalloproteases.	(15)
Liver cancer	Transcriptionally upregulated by RFX5 in HBV-related hepatocellular carcinoma	RBBP6-mediated K48 ubiquitination of FOXP3 promotes glucose metabolism, cell proliferation, migration, invasion, and angiogenesis in HBV-related hepatocellular carcinoma.	(58)

## 11 Role of RBBP6 in therapeutic resistance in cancer

RBBP6 may also be linked to treatment resistance. A study using both *in vitro* and *in vivo* colorectal cancer models showed that silencing RBBP6 enhanced radiosensitivity, whereas its overexpression facilitated radioresistance (31). High expression of RBBP6 has been associated with poor outcomes in colorectal cancer patients. Moreover, elevated RBBP6 expression alone or in combination with mutant p53 correlates with poor survival in CRC patients (72). Cellular studies in colorectal and gastric cancers have shown that knockdown of RBBP6 increases the sensitivity of these cells to radiotherapy, with gastric cancer cells employing the p53 pathway for apoptosis. The G2/M phase is known as the most sensitive phase of the cell cycle to radiotherapy. In gastric cancer, silencing RBBP6 increased G2/M cell cycle arrest. Another study reported that RBBP6 overexpression induces resistance to apoptosis in colorectal cancer cells, which may suppress the therapeutic efficacy of preoperative radiotherapy. This study suggested that RBBP6 expression was a predictive molecular marker for the radiation response and that suppression of RBBP6 could increase the sensitivity of human colorectal cancer cells to radiotherapy (31).

## 12 The role of RBBP6 in alternative cleavage and polyadenylation: implications for carcinogenesis

mRNA splicing and alternative polyadenylation (APA) are critical molecular mechanisms in mRNA processing involved in the posttranscriptional regulation of gene expression. They influence mRNA maturation, stability, degradation, localization, and protein expression, which can influence oncogenes and tumor suppressors, significantly impacting cancer development, progression, and therapeutic response (3, 81). RBBP6 has emerged as a key regulator of mRNA splicing and stability that ultimately affects the expression of oncogenes and tumor suppressor genes, thereby disrupting cellular homeostasis and driving tumor progression (10). It was previously reported that RBBP6 uses its DWNN to associate with nuclear speckles and activate 3’-end mRNA processing. Interestingly, RBBP6, as an essential cleavage and polyadenylation factor, utilizes its C-terminal intrinsically disordered region (IDR) to associate strongly with nuclear speckles and initiate APA events (25). Notably, although the conserved DWNN of RBBP6 is sufficient for pre-mRNA 3′ processing *in vitro*, its IDR-mediated association with nuclear speckles is required for efficient pre-mRNA 3′ processing in cells (25).

Boreikaite et al. (95) used sequence analysis to propose that RBBP6 interacts with the WDR33 and CPSF73 subunits of CPSF to activate pre-mRNA processing in humans. CPSF3 is a component of the cleavage and polyadenylation specificity factor (CPSF) complex, which is critically involved in polyadenylation. CPSF3 has endonuclease activity and recognizes and cleaves pre-mRNA at specific sites during 3’ end processing. A study that directly associated RBBP6 with its role as an APA factor through its E3 ligase activity on CPSF3 to promote carcinogenesis was performed by Lin et al. (3). They provided evidence that RBBP6 mediated K63-linked ubiquitination of CPSF3, which stabilized CPSF3 to regulate APA events. Pharmacological inhibition of CPSF3 with a small-molecule inhibitor (JTE-607) suppressed cell viability and inhibited tumor growth *in vivo*. These data indicate that RBBP6 indirectly sustains elevated MYC expression in glioblastoma stem cells by regulating alternative polyadenylation in a CPSF3-dependent manner (3).

## 13 Role of RBBP6 in cancer metabolism

Metabolic reprogramming, a hallmark of cancer, is a concept whereby cancer cells highly depend on glycolysis for their energy production, despite adequate oxygen levels, to support rapid cell proliferation and malignant behavior (96). Recent studies have reported that RBBP6 promotes glucose metabolism and contributes to cancer progression.

RBBP6 promotes the ubiquitination of JUP, which subsequently contributes to increased glycolysis and promotes metastasis in thyroid cancer (52). Mechanistically, the authors showed that N6-methyladenosine-mediated modification of a circular RNA, circSSU72, promoted its binding to RBBP6, which induced the ubiquitination of JUP (52). Silencing circSSU72 attenuated RBBP6-mediated ubiquitination of JUP, which significantly decreased glucose uptake, lactate production, the extracellular acidification rate, and ATP levels, and JUP knockdown reversed these effects (52).

RBBP6-mediated ubiquitination of FOXP3 enhances glycolysis and accelerates the malignant development of HBV-related hepatocellular carcinoma (58). RBBP6 knockdown restored FOXP3 function and suppressed glucose metabolism, proliferation, and metastasis while promoting apoptosis. Interestingly, RBBP6 is transcriptionally activated by regulatory factor X5 (RFX5), a critical regulator of the expression of MHC class II genes, which are vital for adaptive immune responses (58, 97).

Pancreatic lipotoxicity is a metabolic condition in which excessive lipid accumulation impairs β-cell function. Pancreatic β-cells can be significantly affected by lipotoxicity, leading to cellular dysfunction or death, which can promote type 2 diabetes or aggravate the risk of pancreatic cancer. In a recent study, *in silico* methods were used to predict that RBBP6 ubiquitinates the glucose-responsive transcription factor ChREBP (2). Subsequent work confirmed this prediction by showing that the circular RNA circMlxipl in lipotoxic pancreatic β-cells enhanced the RBBP6-mediated ubiquitination and proteasomal degradation of ChREBP (2). Diminished ChREBP levels significantly impact pancreatic β-cell function and glucose metabolism. Although no direct association has been made regarding RBBP6-mediated ChREBP ubiquitination and proteasomal degradation in cancer progression, ChREBP is itself known to be associated with several types of cancer, including colon cancer and hepatocellular carcinoma, primarily by modulating metabolic pathways (98–100). ChREBP, therefore, influences metabolic reprogramming, as ChREBP knockdown decreases glycolysis and increases oxygen consumption in cancer (101). Finally, p53 plays a pivotal role in regulating glucose metabolism through diverse mechanisms (102), and RBBP6 is known to suppress p53 function. Thus, RBBP6 may indirectly influence glucose metabolism through its interaction with p53.

## 14 Conclusion and future directions

RBBP6 exhibits multifaceted mechanisms to orchestrate its cancer-promoting functions. These functions seem to be embodied primarily by its core molecular functions as an alternative polyadenylation (APA) and splicing factor, and its role as an E3 ubiquitin ligase protein. Intriguingly, both roles seem to intersect in some scenarios where the ubiquitination of a substrate protein leads to the regulation of APA events and promotes carcinogenesis (3).

miRNAs and other noncoding RNAs profoundly affect gene regulation in healthy and disease states. To recognize the crucial role of miRNAs, the Nobel Prize in Physiology or Medicine for 2024 was awarded for their discovery and role in posttranscriptional gene regulation. miRNAs, circRNAs, and lncRNAs exhibit profound regulatory effects on RBBP6, which in turn promotes cancer progression (8, 52, 81, 86). The promoter region of RBBP6 is known to be hypomethylated and activated by multiple oncogenic transcription factors that induce RBBP6 overexpression in colorectal cancer (11). Moreover, aberrant RBBP6 promoter methylation influences miRNA binding to the 3’-UTR of RBBP6, promoting its carcinogenic role in cervical cancer (86). RBBP6 was also reported to trigger the phosphorylation and activation of a critical prosurvival protein, JNK, in cervical and prostate cancers (15, 16).

RBBP6 overexpression is a prognostic and predictive biomarker because it is associated with poor clinical outcomes and poor response to therapy in patients with several types of cancer. Perhaps the most critical interaction of RBBP6 in a procarcinogenic context is its ability to regulate the tumor suppressor p53, which is mutated or inactivated in over 50% of cancers. The therapeutic targeting of the interaction between RBBP6 and p53 is strongly dependent on an understanding of crucial downstream signaling pathways regulated by RBBP6, an accurate structural understanding of the p53 binding domain of RBBP6, and its ability to interact with p53 (56).

The overexpression of RBBP6 strongly correlates with cancer progression in several types of cancer, making RBBP6 a promising immunotherapeutic target. Researchers reported that the RBBP6–specific CTL line suppressed the tumor growth of a human esophageal cancer cell line engrafted in nude mice, providing a strong case for RBBP6 as a tumor antigen for immunotherapy in esophageal cancer patients. Finally, YB-1 is known to interact with programmed death ligand 1 (PD-L1), a well-known checkpoint molecule, suggesting that RBBP6 could play an indirect role in modulating the tumor immune response by ubiquitinating YB-1. Targeting the YB-1/PD-L1 axis enhances chemotherapy sensitivity and boosts antitumor immunity (103). The ability of RBBP6 to ubiquitinate YB-1 raises crucial questions about its possible role in the immune system. However, further studies are needed to examine the role of RBBP6 in regulating the immune tumor microenvironment.

In summary, RBBP6 promotes several hallmarks of cancer. It activates invasion and metastasis through NF-κB signaling, sustains cell proliferation by activating JNK signaling, evades growth suppressors by suppressing p53 and Bax and promoting BCL-2 expression, reprograms energy metabolism by stimulating glycolysis in thyroid cancer, regulates non-mutational epigenetic reprogramming through its activity with noncoding RNAs, and affects cancer cell phenotypic plasticity through maintaining glioblastoma stem cells.

Although RBBP6 critically regulates oncogenic processes, further research is needed to translate these findings into clinical practice. For example, differential targeting of RBBP6 isoforms is known to play contrasting roles in cancer progression. Moreover, it is crucial to further characterize the E3 ubiquitin ligase activity of RBBP6 by identifying other cancer-associated substrates and delineating affected downstream signaling pathways through proteomic analyses and complementary approaches. Probing RBBP6-regulated pathway interactions in critical signaling pathways, such as NF-κB and JNK, under hypoxic, inflammatory, and chemotherapeutic conditions may identify novel anticancer targets. Finally, RBBP6 is a prognostic and predictive biomarker, and validating its use could advance cancer treatment.
